# The price of your soul: neural evidence for the non-utilitarian representation of sacred values

**DOI:** 10.1098/rstb.2011.0262

**Published:** 2012-03-05

**Authors:** Gregory S. Berns, Emily Bell, C. Monica Capra, Michael J. Prietula, Sara Moore, Brittany Anderson, Jeremy Ginges, Scott Atran

**Affiliations:** 1 Department of Economics, Emory University, 1602 Fishburne Drive, Atlanta, GA 30322, USA; 2 Goizueta Business School, Emory University, Atlanta, GA 30322, USA; 3 Department of Psychology, New School for Social Research, 80 Fifth Avenue, New York, NY 10011, USA; 4 Institut Jean Nicod, CNRS-Ecole Normale Supérieure, 29, Rue d'Ulum, 75005 Paris, France

**Keywords:** functional magnetic resonance imaging, sacred values, utility, deontologic, rules

## Abstract

Sacred values, such as those associated with religious or ethnic identity, underlie many important individual and group decisions in life, and individuals typically resist attempts to trade off their sacred values in exchange for material benefits. Deontological theory suggests that sacred values are processed based on rights and wrongs irrespective of outcomes, while utilitarian theory suggests that they are processed based on costs and benefits of potential outcomes, but which mode of processing an individual naturally uses is unknown. The study of decisions over sacred values is difficult because outcomes cannot typically be realized in a laboratory, and hence little is known about the neural representation and processing of sacred values. We used an experimental paradigm that used integrity as a proxy for sacredness and which paid real money to induce individuals to sell their personal values. Using functional magnetic resonance imaging (fMRI), we found that values that people refused to sell (sacred values) were associated with increased activity in the left temporoparietal junction and ventrolateral prefrontal cortex, regions previously associated with semantic rule retrieval. This suggests that sacred values affect behaviour through the retrieval and processing of deontic rules and not through a utilitarian evaluation of costs and benefits.

## 1. Introduction

Sacred values include fundamental religious beliefs, core constructs of national and ethnic identities and moral norms. These values motivate many important individual and group decisions in life. Decisions bounded by them range from purchasing consumer goods such as kosher foods, patronizing Christian businesses, investing in socially responsible mutual funds, to deciding whom to marry. Disagreements over sacred values also contribute to many political and military conflicts and may also underlie some acts of political violence [1,2]. Thus, understanding how sacred values are represented and processed in the human mind has far-reaching implications for policymakers.

By definition, personal sacred values are values for which individuals resist trade-offs with other values, particularly economic or materialistic incentives [3]. The nature of sacred values is, in large part, defined by the way in which individuals engage them in decisions, but virtue theory suggests two very different ways in which sacred values might be processed [4]. Sacred values could be either deontological in nature [5] or they could be utilitarian [6,7]. Deontic processing is defined by an emphasis on rights and wrongs, whereas utilitarian processing is characterized by costs and benefits. Similarly, deontic processing tends to be absolute and independent of outcomes, while utilitarian processing depends on the relative valuation of outcomes. Utility theory has emerged as a normative framework for the latter [8,9], and when applied to decisions over sacred values, suggests that the expectation of consequences for violating these values is a deterrent to certain behaviours [10]. Lexicographic preferences, in which an agent infinitely prefers one thing to another, have also been used to model sacred values within the utilitarian framework [11]. In contrast, the deontic approach suggests that sacred values are derived from rules that circumscribe certain actions independently of expected outcomes or prospects of success, and that we act in accordance with them because they are the right thing to do [3,10].

Until recently, researchers had to rely solely on the decision-makers' self-reports via ratings, rankings or rationale, which are often taken as evidence in favour of one theory or the other. Although insightful, these reports may be influenced by the context of being studied and what are perceived as socially acceptable reasons for doing things. Because it is very difficult to provide realistic outcomes for sacred decisions in an experiment, it is hard to measure behavioural parameters that would allow one to infer the structure of the sacred value decision space. Functional magnetic resonance imaging (fMRI) has emerged as a viable tool to measure brain regions associated with different aspects of decision-making, and the growing literature on the neural correlates of moral judgement has demonstrated that deontic and utilitarian processing are associated with different brain regions [12–17].

While the previous literature has elucidated which brain regions become active when individuals engage either deontic or utilitarian reasoning, the question remains as to how individuals naturally represent sacred values when not forced into a particular framework of decision-making. For example, one might consider the permissibility of killing a human being (the sanctity of human life being a common sacred value) in terms of rights and wrongs or in terms of consequences (e.g. legal sanctions or the effect on the victim's family), and either mode can be imposed by a particular experimental situation. Here, we use fMRI to determine whether sacred values are naturally represented as deontological rules or utilitarian values, unconstrained by a choice.

Clearly, this project presents some methodological challenges. It is not possible, nor desirable to request participants in an experiment to make actual decisions that would violate their values. However, we can use an element of sacredness that captures many of the key characteristics in the laboratory: integrity. Here, integrity refers to an individual's consistency of values and actions. For example, although we cannot (and do not wish to) test whether an individual is willing to kill an innocent human being, we can test their willingness to sign a document that says they would. Although signing such a document does not bind the person to that action, it creates an inconsistency between value and action that signals a loss of integrity. It is reasonable to assume that if something is truly sacred, then an individual would maintain their integrity for that value and not sign such a document. What if they were offered money to sign? It then becomes a trade-off between the monetary gain and the cost in personal integrity. In such a scenario, the amount of money required is one measure of integrity for a particular value, as is their willingness to set a price in the first place.

If sacred values are represented in a utilitarian manner, then prior neuroeconomic research suggests that they should be associated with increased neural activity in brain regions associated with the calculation of utility. Because sacred values are preferred above all else, these values should elicit the highest activity in regions processing utility. The most likely regions include ventromedial prefrontal cortex (VMPFC) [18,19], striatum/nucleus accumbens [20–22] and parietal cortex [23,24]. Alternatively, if sacred values are represented as deontic rules, then brain regions associated with the processing of moral permissibility (rights and wrongs) should show increased activity, with the temporoparietal junction (TPJ) and the MPFC being the most commonly implicated regions [17,25]. Additionally, because rights and wrongs tend to be absolute and stored as rules, deontic processing should also be associated with the retrieval and processing of semantic knowledge. The literature on brain regions involved in semantic retrieval is extensive; however, common themes have emerged with respect to rule retrieval. Several studies have shown that the ventrolateral prefrontal cortex (VLPFC) supports storage or retrieval of semantic forms of stimulus–response contingencies, which are usually referred to as rules [26–30]. Moreover, ‘the VLPFC may be more broadly involved in the retrieval and selection of representations that help to guide and constrain action through stored knowledge’ [31]. Thus, if sacred values are naturally processed in a utilitarian manner, one would expect to see activity in VMPFC, striatum and parietal cortex; or, if they are processed deontologically, one would expect to see activity in the TPJ and VLPFC.

## 2. Methods

### (a) Participants

Forty-three adult participants (see the electronic supplementary material for demographics) took part in the study, which was approved by the Emory University Institutional Review Board. Of these, 11 participated in the experiment outside of the scanner and 32 participated in the scanner. We present behavioural data for all participants and imaging data for those who were scanned. All participants reported good health with no history of psychiatric and neurological disorders and gave written informed consent. Participants received $40 base pay ($20 for those not scanned) and were given the opportunity to earn more money by auctioning their personal values (see §2*b*).

To confirm generalizability of the fMRI data to a broader sample and to examine the relationship between sacred values and group involvement, we also collected data online. An online sample of 391 participants was recruited via the Study Response Project (http://studyresponse.syr.edu) to participate in a survey. Each participant received a compensation of $5. Fifty-seven participants had at least one missing value in the dependent measure, a list of 31 statements. This resulted in an effective sample of *n* = 334 (164 females, 165 males and five did not indicate gender) with ages ranging from 21 to 69 years (*M* = 41.9). This sample was more diverse and more representative of the US population than typical student samples.

### (b) Experimental task

The experimental task was designed to measure the neural responses to statements of personal values that ranged from the mundane to the sacred and test the utilitarian versus deontic representation of sacredness. As a proxy for sacredness, we measured integrity by an individual's willingness to accept real money to sign a document contradicting one's personal values (see the electronic supplementary material for details). The task was divided into four phases (one to three in the scanner). To prevent strategic responding, the instructions for the fourth (auction) phase were given only immediately prior to that phase. First, in the passive phase, participants were presented with value statements phrased in the second person, one at a time. No decision was required. Statements ranged from values that were thought to represent mundane preferences—e.g. ‘You are a dog person’ and ‘You are a Pepsi drinker’—to values describing attributions or acts that were thought to be sacred—e.g. ‘You believe in God’ and ‘You are willing to kill an innocent human being.’ Every statement also had a complement—e.g. ‘You are a cat person,’ ‘You are a Coke drinker,’ ‘You do not believe in God,’ and ‘You are not willing to kill an innocent human being.’ A total of 62 pairs of statements (124 individual statements) were presented in a random order. The purpose of this phase was to record a choiceless response to each statement and see whether utility or deontic systems dominate processing. Next, in the active phase, complementary statements were presented together, and for each pair, the participant had to choose one of the statements. In the third phase, the hypothetical phase, each statement chosen in the active phase was presented with a hypothetical offer of money to disavow the choice they had made in the active phase. For example, if someone previously chose ‘You believe in God,’ then the offer was, ‘Is there a dollar amount that you would accept to disavow your belief in God for the rest of your life?’

In the fourth phase, the auction phase, participants were given the opportunity to sell their answers from the active phase for real money. Using the Becker–DeGroot–Marshak (BDM) auction mechanism, participants were instructed to specify an ‘ask’ price for each of the statements they chose in the active phase [32]. The price could range from $1 to $100. The BDM auction is generally accepted be an incentive-compatible mechanism to reveal an individual's willingness to pay for something. Here, we use it as a willingness to accept. Submitting an ask price of $1, for example, means that the individual is willing to accept any amount of money and is assured of receiving some amount, which, on average, would be $50. In contrast, an ask price of $100 means that there would only be a 1 per cent chance of receiving money. Importantly, they could also opt out of the auction for any or all items, choosing not to alter their answer. Each item was classified based on whether the participant submitted a price in the auction (bid) or opted out of the auction (optout). Submitting a price meant that the participant was willing to exchange this item for money. Opting out meant that the value was non-negotiable or that the amount we offered for the value was not high enough. This provided us with a classification for sacred and non-sacred values.

After all of the ask values were obtained, the participant rolled a pair of 10-sided dice for each of the items for which an ask price was put. If the dice roll was greater than their ask price, they received the value of the dice roll for that item. Their final payment was the average of all items sold and not sold. At the end of the auction phase, the participant received a printout of their chosen statements (active phase), which they did not sell in the auction, and the new statements, which were the complements of the statements sold in the auction. The printout had to be signed. Prior to the auction, participants knew they would have to sign the final document of their personal values. In this manner, the signing of the final document provided an additional incentive to reveal true value. To determine the temporal stability of their personal values, participants repeated the active phase choices through an online survey 6–14 months following their fMRI scan. At that point, they were also asked to indicate how they arrived at their decision: rights and wrongs, costs and benefits or neither. The latter responses were subsequently used to create a functional localizer for deontic and utilitarian processing in the brain.

### (c) Follow-up survey

fMRI participants completed a follow-up survey 6–14 months after their scan session. The purpose was to determine the stability of each person's values and whether their decision for each pair in the active phase was primarily deontic or utilitarian. This was conducted through surveymonkey.com and offered an additional $20 compensation for completion. Twenty-eight of 32 (87.5%) participants completed the survey. The survey repeated the active phase, prompting for a choice between complementary items. Following each choice, the participant was asked to indicate how they arrived at their decision. The following three choices were offered: (i) right and wrong; (ii) costs and benefits; and (iii) neither.

### (d) Neuroimaging data

Neuroimaging data were collected using a 3 T Siemens Magnetron Trio whole body scanner (Siemens Medical Systems, Erlangen, Germany). Functional data consisted of 33 axial slices that were sampled with a thickness of 3.5 mm and encompassing a field of view of 192 mm with an inplane resolution of 64 × 64 (T2*-weighted, TR = 2000 ms, TE = 30 ms). Each participant completed four runs with 62 trials each, whose length depended on participants' decision time (two runs of passive, one run each of active and hypothetical). The auction was done outside of the scanner.

fMRI data were analysed using SPM5 (Wellcome Department of Imaging Neuroscience, University College London) using a standard two-stage random-effects regression model. Data were subjected to standard pre-processing, including motion correction, slice-timing correction, normalization to a Montreal Neurologic Institute (MNI) template brain and smoothing using an isotropic Gaussian kernel (full-width half-maximum = 8 mm). Statistical thresholds were determined based on the estimated smoothness of the second-level contrasts. Using the AlphaSim routine in analysis of functional neuroimages (AFNI), we estimated the combination of height and extent thresholds that yielded a whole-brain false discovery rate (FDR) less than 0.05. Using a voxel-level threshold of *p* < 0.005, the extent threshold that yielded a cluster-level alpha of 0.05 was determined to be *k* ≥ 53. A 40 per cent grey matter mask was applied to all contrasts before using these thresholds.

First, a functional localizer for deontic versus utilitarian processing was obtained from the active phase. Using the responses from the follow-up survey which characterized the mode of processing for each trial, a first-level model consisting of three conditions was created: (i) right/wrong; (ii) cost/benefit; and (iii) neither. Regions involved in deontic processing were identified by the contrast of (right/wrong—cost/benefit), and utilitarian processing regions were identified by (cost/benefit—right/wrong). Second, the regions of interest (ROIs) obtained from the functional localizer were then used to mask the contrasts in the passive phase. For the first-level model of the passive phase, each statement was categorized based on the participant's subsequent choice in the active phase (chosen versus not chosen) and whether they submitted an ask value during the auction (bid versus optout). This created four conditions: (i) chosen/optout; (ii) chosen/bid; (iii) not chosen/optout; and (iv) not chosen/bid. Clearly, items that were not chosen could not be sold, but because the auction was to switch from the chosen to the not chosen item, they were implicitly part of the choice process. One participant was excluded from the analysis because they submitted bids of $1 for all items, and thus no contrasts could be formed. The contrast from the first-level main effect of optout–bid was input into a second-level model and then masked with the (right/wrong—cost/benefit) and (cost/benefit—right/wrong) maps from the functional localizers. Within each ROI, the average activation across subjects was tested for significance with a *t*-test. Third, to identify additional regions that might contribute to sacred values, we performed a whole-brain analysis on the passive phase of optout versus bid by examining the contrast: ((chosen/optout + not chosen/optout)—(chosen/bid + not chosen/bid)). Mean differential activation between optout–bid in ROIs identified from this contrast was also correlated with subject-specific attributes, including subject age, religiosity, liberalism/conservatism and activism.

### (e) Alternative models

To evaluate the possibility of alternative interpretations of the activation patterns, four additional models were tested. Each of these models included a specific aspect of the stimulus as either a condition or covariate in the first-level model, and thus controlled for it as a ‘nuisance’ variable.

— *Legal doctrine*. Given that many sacred values are also represented in legal doctrine, we sought to control for the possibility that participants were simply processing statements as lawful or not. To do this, we removed legality statements into their own category and tested the original model on non-legality statements. If one item of a complimentary pair was illegal either by US or international law, then it was coded as a legality item. For example, ‘You would kill an innocent human being’ and ‘North Korea should be nuked’ were coded as legality items (as well as their complementary statements), while ‘You believe in God’ and ‘You are a Republican’ were coded as non-legal items (as well as their complements) because neither item in those pairs is governed by legal doctrine. A first-level model was created with five conditions: four for the non-legality items (chosen/optout, chosen/bid, not chosen/optout and not chosen/bid) and one for the legality items. Using the ROIs from the original model, we tested for the significance of the main effect of optout–bid for only the non-legality items in a second-level model.
— *Syntax of statement*. A similar procedure was done to test for the effect of the syntax of the item, with those items phrased as ‘You are … ’ removed into their own category, allowing us to test optout-bid on only the ‘You would do … ’ statements.
— *Statement length*. To control for the length of the statement, each condition was modulated by the number of words in the statement, which served as a nuisance regressor.
— *Semantic richness*. Semantic richness (SR) refers to the amount of semantic information contained in, or associated with, a concept in semantic memory [33]. SR has been previously associated with activation in VLPFC [34–36]. To test the possibility that SR may be partially confounded with our measures of sacredness (e.g. ‘God’ has more associations than ‘Pepsi’), we formulated an alternative model that controlled for the SR of the statements.

## 3. Results

**Figure 1.**
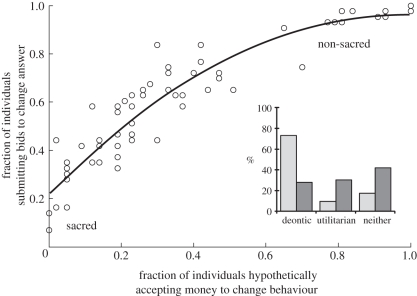
Relationship of auction behaviour to hypothetical solicitation of money. Averaged over participants (*n* = 43), each point represents one pair of personal values. The fraction of individuals submitting auction bids to change their answer for a given pair is plotted against the fraction of individuals who indicated that hypothetically they would accept money to change the corresponding behaviour. These two measurements are highly correlated (adjusted *R*^2^ = 0.87 for quadratic). Items in the lower left (sacred) had a low percentage of individuals willing to accept either hypothetical or real money. These items pertained to the sanctity of human life, especially children. Items in the upper right (non-sacred) had a high percentage of individuals willing to accept both hypothetical and real money to change and represented utilitarian preferences (e.g. Coke versus Pepsi and dog versus cat). Despite this correlation, all of the points lie above the diagonal, indicating a hypothetical bias (participants sold more often than they said they would hypothetically). The mode of decision-making (inset) was significantly different for statements that were not auctioned (optout) versus those that were (bid) (*F*_2,132_ = 58.7, *p* < 0.001). Light grey bars, optout; dark grey bars, bid.

Participants exhibited a wide range of choices with some participants auctioning nearly all of their answers while others very few (mean = 58.6%, range: 8.1–100%). The aggregate distribution of ask values was bimodal, with most being either $1 or optout and a declining tail between the two extremes (see the electronic supplementary material). There was an approximately ordinal and concave relationship between the fraction of individuals submitting bids to change an answer for a particular item and the fraction of individuals who stated they would hypothetically accept money to change their behaviour (figure 1). Follow-up 6–14 months after the initial experiment showed a high degree of stability of sacred values (96.4% consistent), and that sacred values were more stable than non-sacred values (paired *t*_27_ = 7.81, *p* < 0.001). In addition, 73.2 per cent of the time participants selected ‘right/wrong’ as the rationale for choosing a sacred value. In comparison, only 27.8 per cent of the time was the ‘right/wrong’ rationale used to explain choice for items that were not sacred (i.e. those for which a bid was submitted in the auction).

**Figure 2.**
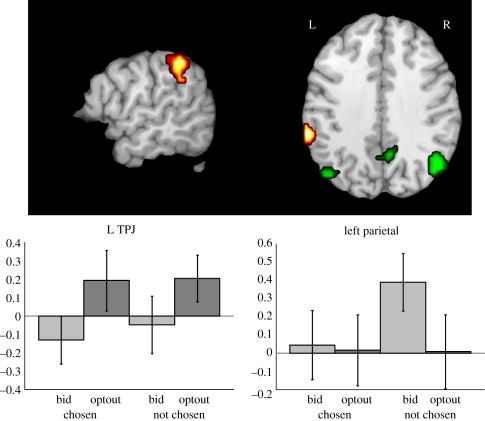
Functional localizer for brain regions with differential activity to deontic (yellow) and utilitarian (green) processing (*p* < 0.005, extent ≥ 53). Regions were classified as deontic when the participant indicated that their choice was based on rights and wrongs, and utilitarian when their choice was based on costs and benefits. These regions were then applied to the passive phase activation in which each statement had been presented individually in the absence of choice. Each statement was categorized based on whether the participant sold a particular personal value during an auction held after the brain imaging session (bid) or opted out of the auction for that value (optout). At the time of imaging, participants did not know that they were going to have the opportunity to sell these values for real money. The left temporoparietal junction (MNI coordinates: −63, −39, 42) showed significantly greater activity for the optout statements than the bid statements (*T* = 3.19, *p* = 0.003), indicating that these were processed in the deontic region. Both the left and right inferior parietal lobule (MNI coordinates: −45, −72, 46 and 48, −66, 35) had the opposite pattern (*T* = 4.09, *p* = 0.001), which was driven primarily by the not chosen bid statements, indicating that these statements were processed in utilitarian regions. Vertical scale on bar graphs is estimated beta values for the individual conditions ± s.e.m. across all subjects.

**Figure 3.**
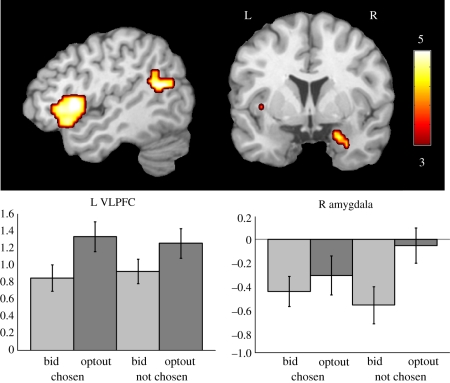
Additional regions identified in which sacred (optout) items resulted in greater activation than non-sacred (bid) items during the passive phase (*p* < 0.005, extent ≥ 53). These included the left ventrolateral prefrontal cortex (L VLPFC) and the right amygdala. Relative to the other three conditions, only the not chosen optout items resulted in more amygdala activation. The latter statements represent the most repugnant items to the individual (those not chosen and not auctioned) and would be expected to provoke the most arousal, which is consistent with the idea that when sacred values are violated they induce outrage [1–3].

To determine whether a stimulus naturally prompted deontological or utilitarian processing, we examined the brain activity during the passive phase. Being the first and unconstrained phase of the experiment, and the only one in which items were presented individually, yielded a window into the natural processing of these values. Before analysing this phase, a functional localizer for deontic versus utilitarian processing was obtained from the active phase. Using the responses from the follow-up survey, which characterized the mode of processing for each trial, regions involved in deontic processing were identified by the contrast of (right/wrong—cost/benefit), and utilitarian processing regions were identified by (cost/benefit—right/wrong). Only the left TPJ was identified as a deontic region, but the orbitofrontal cortex (OFC), and left and right inferior parietal lobules were identified as utilitarian (figure 2). Second, the regions obtained from the functional localizer were then used to mask the contrasts in the passive phase. For the first-level model of the passive phase, each statement was categorized based on the participant's subsequent choice in the active phase (chosen versus not chosen) and whether they submitted an ask value during the auction (bid versus optout). This created four conditions: (i) chosen/optout; (ii) chosen/bid; (iii) not chosen/optout; and (iv) not chosen/bid. The main effect of optout–bid was input into a second-level model and then masked with the deontic and utilitarian maps from the functional localizers. The left TPJ was significantly more active to optout items compared with bid items (*t*_30_ = 3.19, *p* = 0.0034). Both the left and right parietal ROIs were more active for the bid items compared with the optout items (*t*_30_ = 2.59 and 2.04, *p* = 0.015 and 0.05, respectively), but the OFC was not (*t*_30_ = 0.13, *p* = 0.42). To determine if there were additional regions associated with processing sacred values not identified by the functional localizer, we also performed a whole-brain analysis of the passive phase contrast optout-bid, which additionally included the left VLPFC, dorsomedial PFC and right amygdala (figure 3).

## 4. Discussion

These results provide strong evidence that when individuals naturally process statements about sacred values, they use neural systems associated with evaluating rights and wrongs (TPJ) and semantic rule retrieval (VLPFC) but not systems associated with utility. The involvement of the TPJ is consistent with the conjecture that moral sentiments exist as context-independent knowledge in temporal cortex [14,37]. Both the left and right TPJ have been associated with belief attribution during moral judgements of third parties [17]. Our results show that it is also involved in the evaluation of personal sacred values without decision constraints. Thus, one explanation for the reduction in morally prohibited judgements when the TPJ is disrupted by transcranial magnetic stimulation [25] is because disruption impairs access to personal deontic knowledge.

The involvement of the left VLPFC in personal sacred values is also consistent with the conjecture that deontic rules are retrieved and processed as semantic knowledge as opposed to utility calculations. Although the VLPFC has been historically implicated in language function, more recent neuroimaging work has demonstrated that the particular area we identified as processing sacred values is associated with semantic rule retrieval and processing [27–29]. Importantly, the function of the left VLPFC is not restricted to verbal or written rules. In a study of road signs, the anterior division of the left VLPFC was found to be most closely associated with rule retrieval [28]. We observed the same division more active when participants processed sacred values. Similar results were also found in a language-based study of rules, with this region being implicated in top-down retrieval of semantic knowledge [27].

Although activation of the left TPJ and VLPFC for sacred values is consistent with a deontic rule retrieval process, it could be explained by properties of the stimuli, as opposed to how participants processed the stimuli. One possibility is that the items deemed sacred (optout), are governed by legal doctrine and thus the left VLPFC activity simply reflected the retrieval and processing of a legal rule (e.g. it is illegal to kill people). To test this, we created another model in which we added a separate category for items governed by a legal doctrine. An item was coded as a legal doctrine if it or its complement were illegal, either by US or international law (Geneva Conventions). The original analysis was then repeated on the remaining items. Using the original ROIs, we found that the effect of optout–bid was still significant in the left VLPFC (*t*_30_ = 2.15, *p* = 0.040). Thus, even for items not governed by any rule of law (e.g. believing in God), if the individual did not sell it, it was retrieved and processed as a rule. We also tested the possibility that sacred values involve concepts, like God, which have more meanings than mundane concepts such as dogs and cats. SR refers to the amount of semantic information contained in, or associated with, a concept in semantic memory [33] and has been previously associated with activation in VLPFC [34–36]. To test the possibility that SR may be partially confounded with our measures of sacredness, we formulated an alternative model that controlled for the SR of the statements. There was no significant correlation between the SR of the stimulus and the fraction of individuals submitting bids to change their answers (*R*^2^ = 0.045, *p* = 0.053), and when SR was included as a control variable in the passive phase model, significance remained and changed only slightly for the ROIs. This suggests that sacredness was not confounded with SR. We also tested alternative models that controlled for the length and the syntax of each statement, none of which greatly changed the significance of the activations in the ROIs.

Only the left and right inferior parietal lobules showed the opposite activation pattern, with greater activity to the bid versus optout items, which also coincided with cost/benefit decisions (figure 2). This suggests that these regions activate for items that have a measurable utility or value. This is consistent with prior evidence implicating the parietal cortex in utility-based decisions [23,24]. The other region most likely to encode utilitarian values is the VMPFC and striatum [18–22], but we did not observe a significantly greater activation to bid versus optout items in these regions during the passive phase.

The auction mechanism was a unique aspect of our experiment and suggests a new way to quantify sacred values that is not solely dependent on self-report, but there are assumptions behind its use. First, we assume that individuals take the auction seriously. As noted above, signing a document does not bind one to the action that one is signing. It is therefore somewhat surprising that most people did not sell all of their choices. The fact that participants took money for some items and not others suggests that they were adequately motivated to express their preferences through their choices. The upper limit of $100, however, placed a boundary constraint on the auction, which when averaged over all items, yielded a low value per item in expectation (the framing of the auction explicitly instructed participants to value each item in the $1–100 range, and all participants' questions about the auction pertained to how to earn the most money). Although $100 may have been insufficient to buy some answers, this could be true for any amount of money offered. The distribution of bids, however, suggests that this was not the case (see the electronic supplementary material). Although the distribution was dominated bimodally by $1 and optout, the ask values showed a declining frequency towards the $100 boundary, which could be fit by a gamma distribution. This indicates a decreasing marginal exchange value, and a higher upper limit would not have made a significant difference in items that were not auctioned.

**Figure 4.**
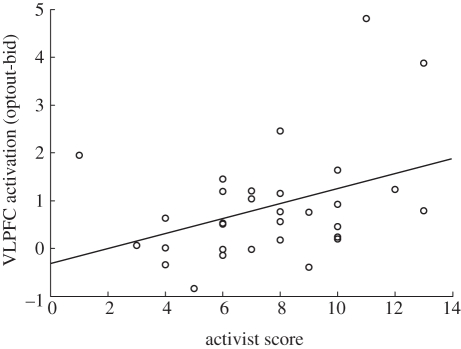
Difference in left ventrolateral prefrontal cortex (VLPFC) activation to sacred items (optout) relative to non-sacred (bid) items as function of each participant's level of involvement in group activities (*n* = 31). The activist score was calculated as the sum of ratings for membership in 10 types of organization. Participants rated their involvement as 0 (do not belong), 1 (inactive member) or 2 (active member) for each organization: religious, sports/recreational, art/music/educational, labour union, political party, environmental, professional, humanitarian/charitable, consumer, other. There was a significant positive correlation between the overall level of organization involvement and the average difference in VLPFC activation to sacred and non-sacred items (*R*^2^ = 0.39, *p* = 0.032). This suggests that individuals who have stronger semantic representations of sacred values are more likely to act on their beliefs.

Our experiment dovetails with a large literature on the neural correlates of moral judgement [12–17]. However, it differs in that it initially measured the natural mode of processing sacred values in a way that was relatively unconstrained by a choice framework. This is particularly important for the scientific study of sacred values, because one cannot ethically place volunteers in real situations that would test such values. However, recent findings in neuroeconomics have demonstrated that ‘choiceless’ brain responses are predictive of future actions [38,39]. Here, we find tantalizing evidence of this for sacred values too. We also found that the difference in VLPFC activation between optout and bid items correlated with the individual's level of involvement in organizations (figure 4). This suggests that neural markers for sacredness extend to real-world decisions of group membership. Moreover, when sacred values were contradicted by their opposites, we observed a significant increase in amygdala activation, which suggests the presence of an arousal response and is consistent with the hypothesized role of emotion, especially negative emotions, when sacred values are violated [1–3,10].

Our results complement existing research in sacred values and may have implications for policymakers [1,2], although further research in conditions that emulate policymaking environments will be required to make the case. Economic, foreign and military policies are typically based on utilitarian considerations. More specifically, it is believed that those who challenge a functioning social contract should concede if an adequate trade-off is provided (e.g. sanctions or other incentives). However, when individuals hold some values to be sacred, they fail to make trade-offs, rendering positive or negative incentives ineffective at best. Our results suggest that individuals naturally retrieve sacred values as deontic rules, not as representations of utility, providing the first neurobiological evidence for what has been previously conjectured [3].
